# Spatial Characterization of Tumor-Infiltrating Lymphocytes and Breast Cancer Progression

**DOI:** 10.3390/cancers14092148

**Published:** 2022-04-26

**Authors:** Danielle J. Fassler, Luke A. Torre-Healy, Rajarsi Gupta, Alina M. Hamilton, Soma Kobayashi, Sarah C. Van Alsten, Yuwei Zhang, Tahsin Kurc, Richard A. Moffitt, Melissa A. Troester, Katherine A. Hoadley, Joel Saltz

**Affiliations:** 1 Department of Biomedical Informatics, Stony Brook University, Stony Brook, NY 11790, USA; danielle.fassler@stonybrookmedicine.edu (D.J.F.); luke.torrehealy@stonybrookmedicine.edu (L.A.T.-H.); rajarsi.gupta@stonybrookmedicine.edu (R.G.); soma.kobayashi@stonybrookmedicine.edu (S.K.); yuwei.zhang@stonybrookmedicine.edu (Y.Z.); tahsin.kurc@stonybrook.edu (T.K.); richard.moffitt@stonybrookmedicine.edu (R.A.M.); 2 Department of Pathology and Laboratory Medicine, University of North Carolina at Chapel Hill, Chapel Hill, NC 27599, USA; alinamh@email.unc.edu (A.M.H.); sarahvan@email.unc.edu (S.C.V.A.); troester@unc.edu (M.A.T.); 3 Department of Genetics, University of North Carolina at Chapel Hill, Chapel Hill, NC 27599, USA; hoadley@med.unc.edu

**Keywords:** TILs, breast cancer, machine learning, computational pathology, risk of recurrence

## Abstract

**Simple Summary:**

The assessment of tumor-infiltrating lymphocytes (TILs) is gaining acceptance as a robust biomarker to help predict prognosis and treatment response. We evaluated TILs in whole-slide images (WSIs) of breast cancer tissue specimens stained with hematoxylin and eosin (H&E) from the Cancer Genome Atlas (TCGA BRCA) and the Carolina Breast Cancer Study (UNC CBCS). Our approach utilized computational pathology to characterize the abundance and spatial distribution of TIL infiltrates in breast cancer WSIs. This work (1) examines the relationship between the global abundance and spatial features of TIL infiltrates with clinical outcomes in order to (2) evaluate their significance as prognostic biomarkers in a multifactorial analysis of progression-free interval in the TCGA BRCA and UNC CBCS datasets. Our findings present a paradigm for pathologists to assess the risk of recurrence in breast cancer by using computational pathology to spatially map, quantify, and interpret TILs in the tumor microenvironment.

**Abstract:**

Tumor-infiltrating lymphocytes (TILs) have been established as a robust prognostic biomarker in breast cancer, with emerging utility in predicting treatment response in the adjuvant and neoadjuvant settings. In this study, the role of TILs in predicting overall survival and progression-free interval was evaluated in two independent cohorts of breast cancer from the Cancer Genome Atlas (TCGA BRCA) and the Carolina Breast Cancer Study (UNC CBCS). We utilized machine learning and computer vision algorithms to characterize TIL infiltrates in digital whole-slide images (WSIs) of breast cancer stained with hematoxylin and eosin (H&E). Multiple parameters were used to characterize the global abundance and spatial features of TIL infiltrates. Univariate and multivariate analyses show that large aggregates of peritumoral and intratumoral TILs (forests) were associated with longer survival, whereas the absence of intratumoral TILs (deserts) is associated with increased risk of recurrence. Patients with two or more high-risk spatial features were associated with significantly shorter progression-free interval (PFI). This study demonstrates the practical utility of Pathomics in evaluating the clinical significance of the abundance and spatial patterns of distribution of TIL infiltrates as important biomarkers in breast cancer.

## 1. Introduction

Our understanding of immune interactions in oncology has evolved considerably since Virchow associated inflammation with cancer [1]. The field of tumor immunology gained further momentum after Coley witnessed tumor regression associated with a Streptococcal skin infection and tumor shrinkage with injection of bacterial products [1,2,3,4,5,6,7,8]. These observations were followed by Ehrlich proposing the concept of host immune responses fighting against cancer and the association of intratumoral lymphocytic infiltration with prolonged survival in cancer patients reported by MacCarty and Mahle [2,9]. In the last 100 years, numerous studies have investigated the relationship of tumor immunosurveillance and patient survival in various types of cancers, which have shown that innate and adaptive immune responses directly and indirectly influence oncogenesis and cancer progression [5,6,8,10,11,12,13].

This study utilizes computational pathology to study tumor immune interactions in digital hematoxylin and eosin (H&E) whole-slide images (WSIs) of breast cancer. Machine learning and computer vision were used to perform Pathomics image analysis to identify tumor regions and lymphocytic infiltrates in breast cancer images. We analyzed breast cancer H&E WSIs from two cohorts, the Cancer Genome Atlas (TCGA BRCA) and Carolina Breast Cancer Study at the University of North Carolina at Chapel Hill (UNC CBCS). Our goal was to investigate the relationship between the abundance and spatial distribution of tumor-infiltrating lymphocytes (TILs) with clinical outcomes in two cohorts that vary greatly in composition. The TCGA BRCA consists of mostly Caucasian patients with larger tumors in advanced stages of breast cancer, which have been aggregated across geographical location. In contrast, the UNC CBCS cohort was designed to study breast cancer in a predominantly younger and African American population in earlier stages of disease since these two groups disproportionately experience worse outcomes.

**Figure 1:**
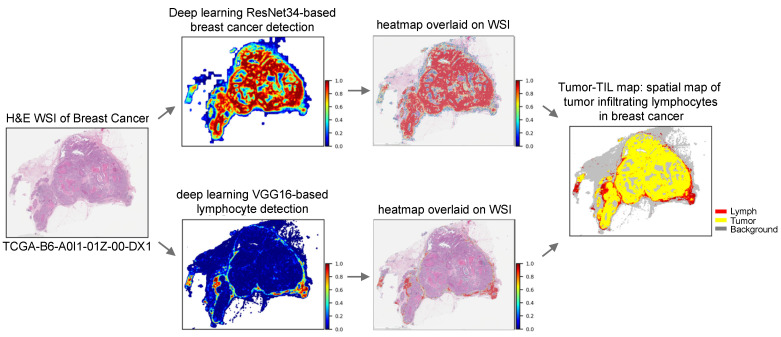
Machine learning and computer vision in computational pathology to spatially map tumor-infiltrating lymphocytes (TILs) in breast cancer. Top panels show tumor detection presented as a spatial probability heatmap to evaluate algorithmic performance (non-tumor tissue colored blue), which is then overlaid on the original H&E WSI. Bottom panels show automated lymphocyte detection presented as a spatial probability heatmap (non-lymphocyte tissue colored blue) and then overlaid on the original H&E WSI. Combining the outputs of tumor and lymphocyte detection generates Tumor-TIL maps to evaluate the abundance and spatial distribution of peritumoral and intratumoral TILs (tumor colored yellow, lymphocytes colored red, and background non-tumor/non-lymphocyte tissue colored gray). This Tumor-TIL map shows the presence of peritumoral TILs with a paucity of intratumoral TIL infiltrates. Image: BRCA TCGA-B6-A0I1-01Z-00-DX1, high-grade breast cancer; cancer detection with ResNet model; lymphocyte detection with VGG16 model.

We previously analyzed several thousands of H&E WSIs across distinct types of cancer from several organ sites [14,15,16] with a first-generation lymphocyte detection model, developed with machine learning and computer vision. The lymphocyte model utilizes convolutional neural networks to identify two or more lymphocytes in tiled 50 square micron image patches to generate spatial maps of lymphocytic infiltrates in 13 different types of cancer [16]. The performance of our model has been further improved through additional training to decrease known false positives and negatives to extend its use to map tumor immune interactions in 23 types of cancer [14,15,16]. A similar approach was used develop a Pathomics tumor segmentation model to delineate tumor regions of breast cancer in H&E WSIs. As shown in Figure 1, the outputs of these machine learning algorithms were combined to generate Tumor-TIL maps in 1021 diagnostic H&E WSIs of breast cancer in TCGA BRCA, which were made publicly available [17].

In this study, the global percentage of intratumoral TILs and the spatial features of TIL infiltrates were correlated with survival and risk of recurrence in TCGA BRCA and UNC CBCS. The global TIL infiltrate percentage in breast cancer regions is based on dividing the number of patches classified as positive for lymphocytes and tumor by the number of total tumor patches to estimate the area of a tumor that is infiltrated by TILs. The computed global TIL infiltrate percentage was correlated with progression-free interval (PFI) and survival in the same manner as the previous study [17].

Pathologists typically describe the constituents, abundance, and spatial distribution of inflammatory infiltrates during histopathologic evaluation of formalin-fixed paraffin-embedded (FFPE) cancer tissue specimens stained with H&E. The magnitude of inflammatory responses is described with terminology such as “minimal, mild, moderate, and severe” or graded as “1+, 2+ or 3+”. Lymphocytic infiltrates are found in many types of malignant neoplasms, including breast, colon, lung, ovarian, and endometrial cancers, as well as melanoma and sarcoma, where their presence has been associated with prolonged survival [5,6,9,12,13,18,19,20]. When the presence of lymphocytic infiltrates is used to classify tumors such as medullary breast cancer, the term “medullary” is used to communicate a relatively favorable prognosis that is based on the presence of lymphocytic infiltrates [5,20,21].

These findings have led to the development and clinical adoption of various forms of immunotherapy in recent decades [19,22,23,24,25,26,27,28,29,30,31,32,33] and a clinical need to assess lymphocytic infiltrates in solid tumors [6,12,25,34,35,36,37,38,39,40,41,42,43,44]. In an effort to uniformly assess and quantify TILs in H&E tissue sections of breast cancer, the TIL Working Group of the International Immuno-oncology Biomarkers Working Group published guidelines for pathologists to score the percentage of stromal TILs (sTILs) to go beyond qualitative descriptions [12,43]. Pathologists are instructed to manually assess sTILs in breast cancer in H&E tissue specimens through a series of steps that include identification of cancer-associated stroma within a tumor region, discerning the type of inflammatory infiltrate, and reporting the percentage of cancer-associated stroma infiltrated by mononuclear lymphoplasmacytic immune cells [12,43,45,46]. Intraepithelial TILs and sTILs in direct contact with cancer cells are excluded.

Several studies have clinically established TILs as a robust prognostic biomarker in triple-negative breast cancer (TNBC) and Her2+ breast cancer [42,47,48,49,50,51,52], which endorse the need to incorporate TILs as part of standard clinical histopathologic examination of TNBC [53,54,55,56]. TILs have also been evaluated in breast cancer as a predictive biomarker in the adjuvant and neoadjuvant settings leading to interest in using TILs to monitor treatment response [7,12,40,42,43,45,46,47,48,54,55,56,57]. The recognized importance of TILs in breast cancer will most likely require TILs to be reported in solid tumors of the skin, lung, gastrointestinal tract, and gynecologic tract as well [12,40]. However, there are pitfalls and challenges associated with the assessment of sTILs that may impact the prediction of outcomes, which are primarily due to the heterogeneity of inflammatory infiltrates and their spatial distribution in breast cancer [40,58].

Mapping TILs in thousands of breast cancer WSIs has revealed a fascinating spectrum of global spatial patterns of lymphocytic immune responses that is not readily evident during traditional light microscopy. Our computational approach that generates Tumor-TIL maps is designed to help interpret global tumor immune interactions at the tissue level. We designed our approach to provide an immediate global view of the relative abundance and spatial heterogeneity of TIL infiltrates to complement and support the tissue/cellular-level evaluation of sTILs endorsed by the TIL Working Group. By providing tissue-level insights with visual maps that accentuate TIL infiltrates in breast cancer regions, our approach may help minimize sampling and observer variability.

We utilized Tumor-TIL maps in TCGA BRCA and UNC CBCS to investigate the clinical significance of TIL infiltrates in spatially distinct geographical regions of breast cancer. We adapted concepts and terminology from ecology to characterize the magnitude and presence of spatial = distribution of (1) intratumoral TIL infiltrates (forests), (2) peritumoral TILs at the invasive margin, (3) tertiary lymphoid aggregates beyond the vicinity of the leading edge of the tumor, and (4) immune cold regions (deserts). Univariate and multivariate analyses in the TCGCA BRCA and UNC CBCS cohorts were used to determine the relationship between the abundance and spatial features of TIL infiltrates, cancer progression, and survival. This work presents a simple Pathomics approach to clinically interpret the abundance and spatial distribution of TILs as biomarkers in breast cancer to potentially predict tumor progression to identify at-risk patients and support the assessment of sTILs in breast cancer.

## 2. Materials and Methods

### 2.1. Datasets and Algorithms

Two independent cohorts, containing demographic, hormone receptor status, and molecular subtype data, were used in this study. Specifically, high-resolution H&E WSIs of breast cancer tissue from the TCGA BRCA [59] and the UNC CBCS [60,61,62,63,64,65,66,67,68] cohorts, as described in Table 1.

#### 2.1.1. The TCGA BRCA Dataset

The TCGA BRCA cohort contains 1098 patients with 1021 diagnostic WSIs of histologic breast specimens. For this study, we excluded patients if diagnostic H&E WSIs were flagged by a pathologist as having scant tumor or significant slide preparation artifact. Samples with normal-like subtype were also excluded, bringing our study cohort to 934 samples. All patients were classified by PAM50 subtypes (Basal: 167, Her2: 76, LumA: 489, LumB: 212) and IHC-derived estrogen receptor status (Negative: 197, non-Negative: 692) [69,70]. Patient stage at primary diagnosis was also recorded (Stage I: 162, Stage II: 545, Stage III: 213, and Stage IV: 15). For survival analysis, we utilized Progression-free interval (PFI) [71] to study 117 events after filtering the cohort.

#### 2.1.2. The UNC CBCS Dataset

The UNC CBCS Phase 3 cohort contains 2998 cases with 1138 diagnostic WSIs from representative blocks selected by a pathologist and associated follow up recurrence and survival data. The date of diagnosis, stage, and recurrence were abstracted from medical records. Date of death data is limited as patients were diagnosed between 2008 and 2013 and are followed by medical record. The UNC CBCS diagnostic WSIs were processed with the same machine learning algorithms that were used to analyze the TCGA BRCA WSIs. Cases with scant tumor, slide preparation artifacts, and normal-like subtype samples were excluded, leaving 1081 cases in the UNC CBCS cohort. Samples were classified by PAM50 subtypes (Basal: 245, Her2: 82, LumA: 553, LumB: 201) and immunohistochemistry (IHC)derived estrogen receptor status (Negative: 249, non-Negative: 831). Patient stage at the time of diagnosis was also recorded (Stage I: 417, Stage II: 499, Stage III: 138, Stage IV: 27).

#### 2.1.3. Selection of Validation (“TIL-Sensitive”) Cohort

Initial analyses of the association of TIL infiltrate percentage with patient outcomes in the TCGA BRCA cohort showed varying prognostic power based on molecular subtype and estrogen hormone receptor status. Given that the cohort composition between TCGA and UNC CBCS is significantly different in terms of tumor stage, patient race, and PAM50 subtype, we sought to ensure a like-to-like comparison of patient cohorts. Using this information, we subsampled a validation cohort of patients from the overall UNC CBCS cohort who were likely to be “TIL sensitive”. For this purpose, we created a cohort that included patients with negative estrogen hormone receptor status by IHC expression and/or patients in the PAM50 LumB and Her2 molecular subtypes (TCGA *n* = 430, UNC CBCS *n* = 481), as shown in Table 1.

### 2.2. Machine Learning Computational Pathology Algorithms

Tumor and lymphocyte detection in H&E WSIs of TCGA BRCA and UNC CBCS were performed by our previously developed machine learning algorithms [15,17], as shown in Figure 1 [15,17]. Breast cancer tumor detection utilizes a ResNet34 model that was previously trained, tested, and validated on breast cancer images from the TCGA and the Surveillance, Epidemiology, and End Results Program of the National Cancer Institutes (NCI SEER) [17]. We spatially mapped lymphocytes by utilizing our VGG16 model that was trained, tested, and validated with images from multiple tumor types [15]. The models process tiled image patches of WSIs with specified patch size for each algorithm, determined by optimizing algorithmic performance. Tumor detection classifies 87.5 × 87.5 µm^2^ patches (equivalent to 350 × 350 square pixels at 40× magnification) and outputs the prediction as a probability, whereas the lymphocyte model classifies 50 × 50 µm^2^ patches (equivalent to 200 × 200 square pixels at 40×) to predict whether a patch contains 2 or more lymphocytes.

#### 2.2.1. Generation of Composite Tumor/TIL Maps

**Figure 2:**
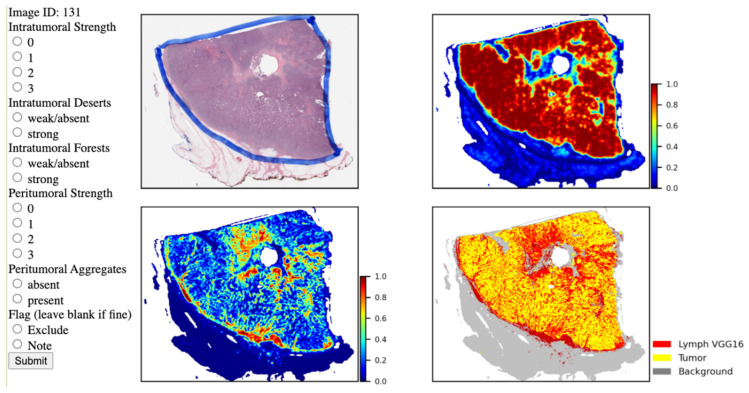
Scoring interface to characterize spatial features of TIL infiltrates in breast cancer. Composite Tumor-TIL maps of H&E WSIs of breast cancer provide the ability to estimate the abundance and spatial distribution of TILs in a straightforward manner. Tumor-TIL maps are depicted as 4-panel composites containing low-resolution H&E WSI (**upper left**), tumor probability heatmap (**upper right**), lymphocyte probability heatmap (**lower left**), and the Tumor-TIL map (**bottom right**). Tumor and lymphocyte probability maps use a color scale to indicate probability from 0 (blue) to 1 (red). In the Tumor-TIL map, yellow represents tumor, red depicts lymphocytes, and non-tumor/non-lymphocyte patches are represented as gray background tissue. Observers use this interface to characterize the magnitude of intratumoral and peritumoral TIL infiltrates on a scale of 0 (none/absent) to 3 (marked). The presence of large intratumoral aggregates (forests), immune cold areas devoid of TILs (deserts), and tertiary lymphoid aggregates are indicated on the left panel. In this example, case 131, intratumoral strength was graded as 3 with weak/absent deserts and strong forests, peritumoral strength was graded as 3, and tertiary peritumoral aggregates as absent. Poor quality images and cases where the algorithms did not properly generate a Tumor-TIL map were flagged and excluded.

As shown in Figure 1, the predicted results for each patch are represented as pixels that are stitched together to generate probability heatmaps to identify cancer regions and lymphocytic infiltrates. The heatmaps were then binarized so that patches with probabilities ≥50% were considered positive and <50% considered negative. The binarized patch-level predictions are ultimately combined in a post-processing step to create Tumor-TIL maps, where yellow represents tumor, red depicts lymphocytes, and non-tumor/non-lymphocyte patches are represented as gray background tissue. We utilized Tumor-TIL maps to identify and grade the abundance of intratumoral TILs, peritumoral TILs, and tertiary lymphoid aggregates for each H&E WSI. A four-panel composite image is generated for each case in the TCGA and UNC CBCS cohorts, which contains a low-resolution image of the H&E WSI, tumor probability heatmap, lymphocyte probability heatmap, and the Tumor-TIL map, as shown in Figure 2.

#### 2.2.2. Calculation of Percent Infiltration

For each patient, TIL infiltrate percentage was calculated as the number of predicted patches that were classified as positive for tumor and lymphocyte divided by total number of cancer patches after scaling and alignment of the outputs from the two models. Survival analyses were performed by scaling the percent infiltration to have a variance of 1. Patients were then categorized as TIL-high or TIL-low based on the mean TIL infiltrate percentage for their respective cohort, as shown in Table 1. Supplemental Figure S2 shows how TIL infiltrate percentage stratifies the patients in the UNC CBCS dataset with respect to molecular subtypes, estrogen receptor status, and stage of disease.

#### 2.2.3. Spatial Feature Scoring

The Tumor-TIL maps revealed fascinating and unique global spatial patterns of the distribution of TIL infiltrates in each WSI of breast cancer, as shown in Figure 2 and Supplemental Figure S1. To characterize these patterns, concepts and terminology were adapted from ecology to describe high-level spatial features of tumor immune interactions that were observed in each case. Candidate features were identified through visual inspection of 1000+ Tumor-TIL maps by two pathologists and one MD/PhD student. Spatial features were defined and graded by the criteria shown in Table 2 by three independent observers at the graduate student trainee level, who were trained by the study pathologists.

**Figure 3:**
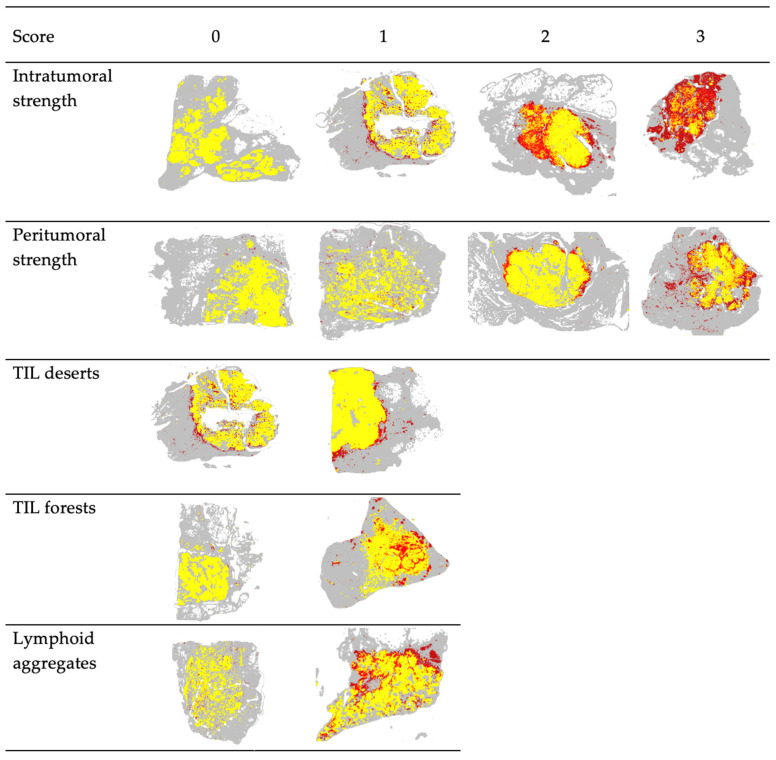
Representative Tumor-TIL maps from TCGA BRCA demonstrating the scoring paradigm for spatial features of TIL infiltrates. Scores of 0, 1, 2, and 3 correspond to terminology used by pathologists for grading, such as minimal, mild, moderate, and severe and/or 1+, 2+, and 3+. Detailed descriptions for scores (columns) for each category (rows) are found in Table 2. Red depicts lymphocytes, yellow depicts tumor regions, and gray represents non-tumor and non-lymphocyte background tissue regions.

A web interface was developed to record characterization of the spatial features of Tumor-TIL maps, as shown in Figure 2. The web tool was also used to flag or exclude cases with poor tissue quality or suboptimal algorithmic performance. The goal was to characterize the abundance and spatial distribution of intratumoral and peritumoral TILs, as well as tertiary lymphoid aggregates in the surrounding tissue microenvironment beyond the invasive margin. Figure 3 depicts how we evaluated *intratumoral strength, intratumoral forests, intratumoral deserts, peritumoral strength, and lymphoid aggregates*. Supplemental Figure S1 shows representative Tumor-TIL maps demonstrating variations in TIL deserts, forests, and lymphoid aggregates. Table 2 provides a description of the criteria used to score the spatial features of TIL infiltrates. All three independent observers used the web tool to score all “TIL-sensitive” cases from the TCGA BRCA and UNC CBCS cohorts. Consensus scores were generated for each feature on each image by taking the median value of the three individual scorers for each feature.

### 2.3. Assessment of Outcomes in Patient Studies with Computed TIL Infiltration

**Figure 4:**
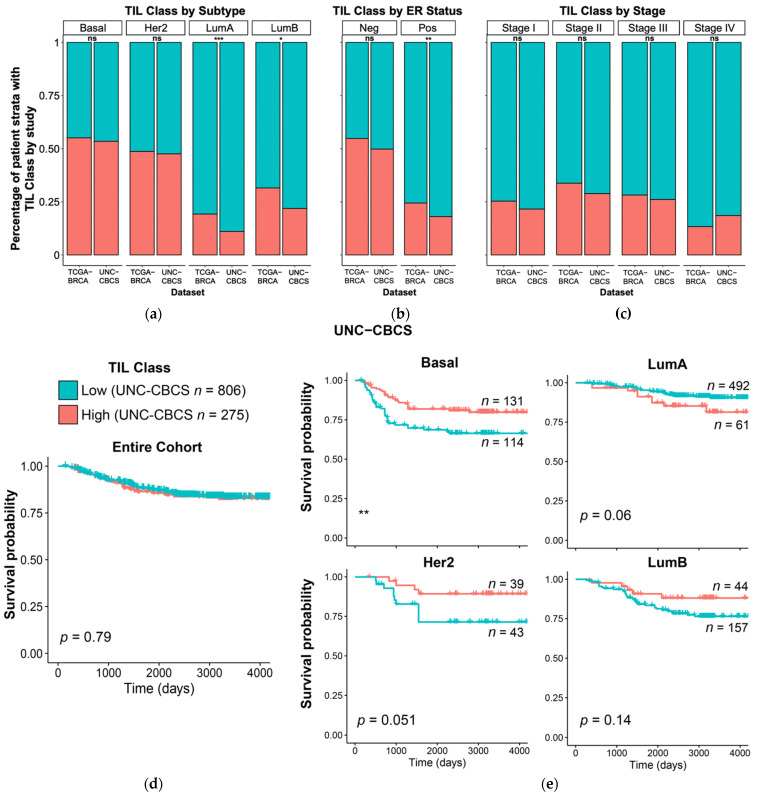
Comparison of computed intratumoral TIL infiltrate percentage in TCGA BRCA and UNC CBCS and relationship to progression-free interval (PFI). The median TIL infiltrate percentage was computed for each study to distinguish cases as high or low TIL class; percent infiltration was determined using VGG TIL detection algorithm. (**a**–**c**) Percentage of patient strata classified as high and low TILs is shown for the TCGA BRCA and UNC CBCS studies grouped by (**a**) PAM50 molecular subtype, (**b**) estrogen receptor (ER) status, and (**c**) tumor stage. Blue denotes TIL class Low and Red denotes TIL class High. (**d**,**e**) Kaplan–Meier plots to show disease progression in the UNC CBCS cohort after dividing patients into high and low TIL classes around the mean TIL infiltrate percentage for the (**d**) entire UNC CBCS cohort and (**e**) cases split by PAM50 molecular subtype. Log-rank test was used to assess survival differences. *** = *p* < 0.001, ** = *p* < 0.05, * = *p* < 0.01.

We first sought to replicate our previous findings by using progression-free interval (PFI) instead of overall survival (OS) [71]. PFI measures the length of time that a patient has no tumor-associated event. An event is registered with progression of disease, local recurrence, distant metastasis, new primary tumors (all sites), or death without new tumor event. PFI is a preferable clinical metric due to the relatively short follow up times for patients in the TCGA BRCA cohort. This method of tracking clinical performance is the same metric utilized in the UNC CBCS, which permitted external validation of comparable metrics. Kaplan–Meier analyses were performed to show how computed TIL infiltrate percentage is useful for predicting survival stratified by molecular subtype in the UNC CBCS dataset, as shown in Figure 4.

### 2.4. Evaluating Risk with Spatial Feature Inclusion

Each consensus spatial feature was assessed for interrater agreement using Fleiss’ Kappa and then used in a univariate Cox Regression model to identify whether the presence of a particular feature increased or decreased risk of recurrence, as shown in Supplemental Figure S3. Despite prognostic impact in the TCGA BRCA cohort, tertiary lymphoid aggregates were excluded from “high-risk features” scoring due to low interrater agreement (κ = 0.37). A Fleiss’ Kappa score of 0.4–0.6 was considered moderate agreement; 0.61–0.80, substantial; 0.81–1.0, almost perfect agreement; below 0.5, unacceptable for downstream analysis [72]. Spatial feature scores that increased risk of recurrence were categorized as “high-risk feature scores”. Patients were then clinically stratified based on the presence of two or more high-risk features and grouped into high-risk and low-risk cohorts.

### 2.5. Statistical Tests

For all *p*-value representations throughout this study, *** = *p* < 0.001, ** = *p* < 0.05, * = *p* < 0.01. Survival experiment *p*-values were generated using log-rank test for Kaplan–Meier analysis or Cox Regression Analysis. Cohort composition differences in Table 1 and Table 2, as well as the bar graphs in Figure 4a were generated using chi-squared test for independence.

## 3. Results

### 3.1. Cohort and Lymphocyte Infiltration Characteristics

We extended our previous analyses of TILs in the TCGA BRCA cohort to study TIL infiltrates in the UNC CBCS cohort of breast cancer patients from North Carolina who were diagnosed from 2008 to 2013. In comparing the UNC CBCS cohort to the TCGA BRCA cohort that contains more aggressive subtypes, the UNC CBCS cohort has a distribution of PAM50 molecular subtypes across different stages of breast cancer that is representative of population-based sampling. Further, UNC CBCS tissue samples reflect the diversity of race and age that were a product of its randomized recruitment design. Significant differences between the TCGA BRCA and UNC CBCS cohorts include race, disease stage, and distribution of PAM50 molecular subtype, as shown in Table 1.

The UNC CBCS patient population was 53.4% African American (vs. 15% in TCGA). The UNC CBCS cohort also has a larger percentage of lower stage tumors, particularly stage I (38.6% vs. 17.3% in TCGA), in comparison to the TCGA BRCA cohort that consists of later stage tumors than the general population. These differences in cohort composition resulted in having complementary datasets. For both cohorts, we calculated TIL infiltrate percentage and grouped patients into high and low TIL infiltration classes around the median. The median percent infiltration for TCGA BRCA was 3.67% (min: 0, max: 64.23) and 1.82% (min: 0, max: 88.90) in the UNC CBCS cohort. The mean TIL infiltrate percentage was 7.56% in TCGA BRCA and 5.55% in UNC CBCS, as shown in Figure 4. The proportion of cases that were classified as TIL class Low was 69.3% in TCGA in comparison to 74.6% in UNC CBCS.

We also characterized differences in TIL infiltrate percentage in the UNC CBCS cohort with respect to molecular subtypes and estrogen receptor status. Using chi-squared analysis, the percentage of patients categorized as TIL class High and TIL class Low did not significantly differ between the TCGA BRCA and UNC CBCS datasets in the Basal, Her2, and ER hormone receptor negative cohorts. Figure 4 also shows that TIL infiltration class by tumor stage was also consistent between study populations, indicating that study level differences will not confound the evaluation of the impact of TIL infiltration on PFI.

### 3.2. Computed Lymphocyte Infiltration from Tumor-TIL Maps Strongly Predicts PFI and Survival

The percent infiltration in the TCGA BRCA and UNC CBCS cohorts were significantly associated with increased PFI in multivariate analysis with PAM50 molecular subtype and tumor stage, as shown in Figure 4. When UNC CBCS cases were split into TIL class High and TIL class Low, we did not observe a difference in length of progression-free interval. However, TIL class High was associated with longer survival in basal, Her2, and LumB molecular subtypes in the UNC CBCS cohort when stratified by PAM50. The forest plots of multivariate Cox proportional Hazard Ratio (HR) show that percent infiltration as a continuous variable is correlated with prolonged progression-free interval in the UNC CBCS cohort in all molecular subtypes except LumA and stages II-IV.

**Figure 5:**
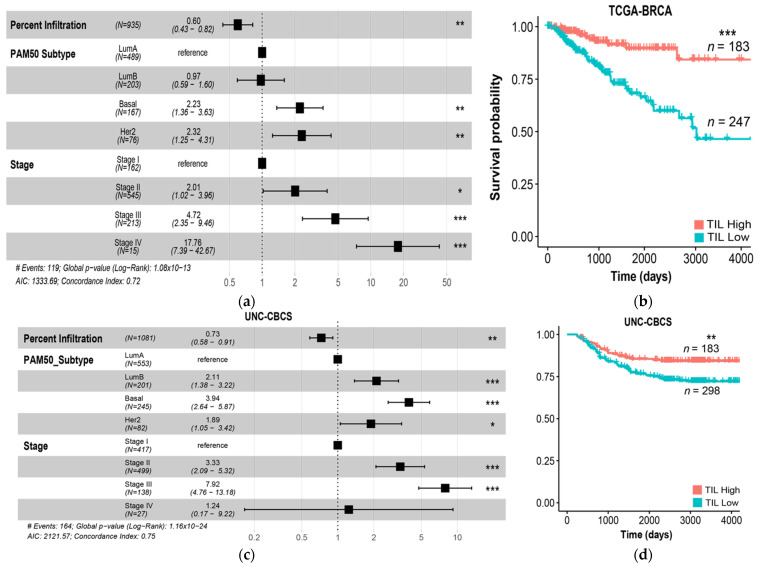
TIL infiltration remains strongly predictive of survival across TCGA BRCA and UNC CBCS. (**a**) Forest plots of hazard ratios (estimates and 95% CIs) from a multivariate Cox proportional hazards model of progression-free interval (PFI) incorporating PAM50 subtype and tumor stage. Vertical dotted line indicates a hazard ratio of 1. TIL infiltrate percentage ranges from 0% to 64.2% with SD of 10.4%. TIL infiltrate percentage is scaled by SD for all Cox Regression Analyses. (**b**) Kaplan–Meier survival analyses of TCGA BRCA patients after splitting into high and low TIL classes around the mean percent infiltration. (**c**) Forest plot of hazard ratios from a multivariate Cox proportional hazards model of PFI in the UNC CBCS including PAM50 subtype and tumor stage. TIL infiltration was calculated as a continuous variable with a range of 0% to 88.9% and scaled by SD (0.1), AIC: 2121.57. The dotted line indicates a hazard ratio of 1. The Concordance Index shows how much variance of risk is explained by the model, where 1 encapsulates all risk whereas 0.5 is random. (**d**) Kaplan–Meier survival analyses after dividing UNC CBCS patients into high and low TIL infiltration groups around the mean TIL infiltrate percentage. *** = *p* < 0.001, ** = *p* < 0.05, * = *p* < 0.01.

To further compare the two study populations, we defined TIL responsive subsets defined as estrogen receptor negative by IHC or PAM50 molecular subtype of LumB or Her2, as shown in Figure 5. In predicted TIL-sensitive cases in both the TCGA BRCA (*n* = 428) and UNC CBCS (*n* = 491) cohorts, both the continuous TIL infiltration and categorical TIL status were robustly associated with outcomes. (Figure 5b,d). TIL class High was significantly associated with increased survival.

### 3.3. Spatial Feature Interrater Agreement and Feature Correlation

Once we annotated the spatial features listed in Table 2, we assessed interrater agreement, shown in Supplemental Figure S2. We measured the reliability of the agreement between observers by using Fleiss’ kappa and show moderate to substantial agreement across all categories, with overall kappa scores ranging from 0.56 to 0.68. We note that there is slightly higher agreement for all five spatial features in TCGA BRCA compared to UNC-UNC CBCS, where the largest difference is observed in identifying and grading the presence of tertiary lymphoid aggregates. We correlated spatial metrics and percent infiltration to observe strong correlations between the magnitude of intratumoral TIL infiltrates and global TIL infiltration percentage.

### 3.4. Relationship between Percent TIL Infiltration and Spatial Features

The distribution of spatial feature scores differed between the “TIL sensitive” subsets of the TCGA BRCA and UNC CBCS cohorts, as seen in Table 3. Spatial features describing TIL infiltrates were positively correlated with percent TIL infiltration, whereas TIL deserts (the absence of TILs) were negatively correlated with percent TIL infiltration. The percentage of cases from the TCGA cohort with high intratumoral strength (scores of 2 or 3) was higher than the UNC CBCS cohort, which was also evident in the percentage of cases with intratumoral TIL forests. The percentage of cases with intratumoral TIL deserts peritumoral lymphoid aggregates was also higher in the UNC CBCS cohort. Peritumoral strength was comparable between cohorts.

### 3.5. Presence of Two or More High-Risk Spatial Features Is Associated with Poor Prognosis

Table 4 shows how graded spatial features of TIL infiltrates were grouped to derive high-risk features. For example, intratumoral strength categories represent intratumoral TIL abundance. Each consensus feature was used in a univariate model to identify whether its presence alone increased or decreased risk. Table 4 shows that increasing intratumoral and peritumoral strength appears to be associated with longer PFI, even though these associations were not significant in both cohorts. However, statistically significant associations showing how immune cold TIL deserts were associated with two times the risk of progression. TIL forests were found to be associated with decreased risk or recurrence in the TCGA BRCA cohort. In the UNC CBCS cohort, only the highest level of peritumoral strength was significantly associated with PFI in univariate analyses.

These trends and statistically significant findings were used to describe “high-risk features,” which include low intratumoral strength (score of 0 or 1), presence of TIL deserts (score of 1), absence of TIL forests (score of 0), low peritumoral strength (score of 0 or 1), and absence of lymphoid aggregates (score of 0). Increasing the number of high-risk features was associated with increased risk of recurrence. We utilized having two or more high-risk features clinically stratify patients into high-risk and low-risk groups to predict risk of progression in the TCGA BRCA and UNC CBCS cohorts.

**Figure 6:**
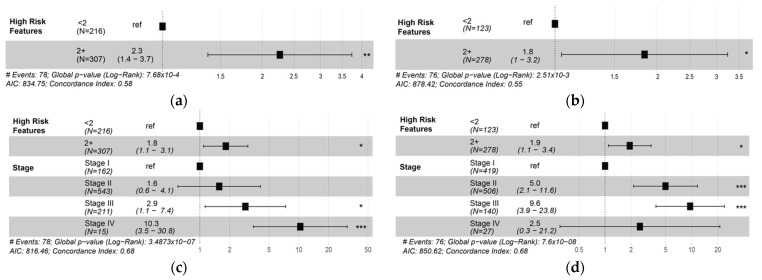
The presence of two or more ‘high-risk’ spatial features provides independent prognostic information. High-risk feature scores include low intratumoral TILs (scores of 0 or 1), absence of TIL forests (0), presence of immune cold TIL deserts (1), and low peritumoral TIL scores (0 or 1). (**a**,**b**) Forest plots of hazard ratios (estimates and 95% CIs) from a univariate Cox proportional hazards model of progression-free interval (PFI) comparing cases with two or more high-risk features (2+) to those with ≤1, as shown in (**a**) TCGA and (**b**) UNC CBCS. (**c**,**d**) Forest plots of hazard ratios (estimates and 95% CIs) from a multivariate Cox proportional hazards model of progression-free interval (PFI). The dotted line indicates a hazard ratio of 1, where (**c**) TCGA and (**d**) UNC CBCS. *** = *p* < 0.001, ** = *p* < 0.05, * = *p* < 0.01.

Figure 6 shows that two or more high-risk spatial features were associated with decreased PFI in both TIL-sensitive TCGA BRCA and UNC CBCS cohorts. Our observations are consistent with what was observed when using computed TIL infiltrate percentage, as shown in Figure 5. The addition of continuous *percent infiltration* to the models shown in Figure 6c,d did not result in a significant increase in the explanation of risk in either cohort, as shown in multivariate analysis in Supplementary Figure S3.

## 4. Discussion

Our pan-cancer analyses of mapping lymphocytes in thousands of H&E WSIs from different types of cancer revealed the exquisite diversity of the global abundance and spatial patterns of TIL infiltrates [73]. As shown in Figure 3 and Supplemental Figure S1, lymphocytic immune responses in cancer are highly complex and heterogeneous within the spatial context of microenvironmental geography in cancer tissue specimens. We previously investigated the spatial architecture of TILs with agglomerative clustering, which was correlated with patient survival in multiple cancer types [16]. At that time, we categorized the spatial patterns of immune responses in TIL maps alongside corresponding H&E WSI by borrowing terminology used to describe peritumoral lymphocytic responses in malignant melanoma [16]. After pairing the ability to map the distribution of lymphocytes with breast cancer tumor segmentation, this work characterizes the spatial features of TIL infiltrates for use alongside computed intratumoral TIL infiltrate percentages.

Combining tumor and lymphocyte detection to quantify TILs with computational pathology is a novel approach that allowed us to confirm that greater intratumoral TIL infiltration is correlated with increased overall survival (OS) in breast cancer in the TCGA BRCA cohort. Stratifying by molecular subtype showed an even more pronounced effect and statistically significant relationship between TIL infiltrate percentage and survival within the Her2 and Luminal B subsets. However, straightforward calculation of TIL infiltrate percentage does not capture the nuances of spatial distribution, so we decided to revisit first principles by exploring whether we could ascertain the clinical significance of the various types of spatially distinct TIL infiltrates that are evident in Tumor-TIL maps.

Analyzing TILs in the H&E WSIs from the UNC CBCS cohort with the same methodology that was used for the TCGA BRCA dataset provided a tremendous opportunity to fundamentally ascertain whether the spatial features of TIL infiltrates have any clinical significance. Before we characterized spatial features, Tumor-TIL maps were used to compute global intratumoral TIL infiltration to confirm previous observations about the prognostic impact of TIL infiltrates stratified by molecular subtype and estrogen receptor hormone status. Despite the differences in the TCGA BRCA and UNC CBCS cohorts in terms of the distribution of age, race, disease stage, and distribution of PAM50 molecular subtypes of breast cancer, many substrata showed similar overall TIL infiltration characteristics, as shown in Figure 4. This supports the use of TILs as a biomarker to predict survival in breast cancer patients with similar clinicopathologic characteristics since this relationship was present when comparing two very different cohorts. Subsampling a TIL-sensitive cohort from UNC CBCS cohort based on previous analyses of TCGA BRCA to study the presence of high-risk spatial features alongside TIL infiltrate percentage lends support to the concept of potentially stratifying patient care and surveillance with respect to risk of recurrence.

As stated previously, the visual assessment of TILs is challenging in cancer-associated stromal regions, which can impact scoring sTILs and predicting outcomes on a case-by-case basis. The primary source of scoring variability was attributed to the heterogeneity of the global distribution of TIL infiltrates in breast cancer [40,58], which is clearly evident when viewing conceptually simple Tumor-TIL maps. Additional factors leading to observer variability include scoring sTILs beyond the boundary of the tumor, minimal cancer-associated stroma, and distinguishing TILs in mixed immune infiltrates. In order to improve consistency, evaluating multiple areas and averaging percentage scores of sTILs, and the use of reference images have been proposed, alongside growing interest in using computational image analysis to evaluate TILs in breast cancer [16,40,58,74,75,76,77,78,79].

Multiple studies have shown that machine learning algorithms can be used to quantify TILs in cancer, including TNBC [40,74,75,77,80]. As we march towards precision medicine, methods that perform tumor segmentation, subclassification of tissue compartments (e.g., neoplastic, dysplastic, and normal epithelium, stroma, and necrosis), and nuclear segmentation and classification have been developed. However, the current limitation is validation since it is extremely cost, time, and resource prohibitive to evaluate the performance of algorithms in correctly identifying and classifying every cell in an image across hundreds of thousands of cells per WSI. Nonetheless, algorithms have been developed that can compute the number of sTILs per the TIL Working Group [74,75,77,80]. We have also developed a method to segment and classify tumor cells, lymphocytes, and stroma (non-tumor and non-lymphocytes) in breast cancer to extend the functionality of Tumor-TILs analyses to identify salient regions to compute sTILs [73,81].

The premise of this study is computationally and conceptually simple in comparison. Since Tumor-TIL maps are useful for quantifying global intratumoral TILs and identifying spatially distinct TIL infiltrates at the tissue level, we investigated the clinical significance of these computed parameters as Pathomics biomarkers in breast cancer. Our downstream analyses are also straightforward since we divide the cohorts into TIL class High and TIL class Low by using the mean value of TIL infiltrate percentage. Therefore, we present this Pathomics workflow to (1) demonstrate how easily additional datasets of breast cancer WSIs can be processed to (2) compute intratumoral TIL infiltrate percentages, (3) characterize tissue-level spatial features of TIL infiltrates, and (4) correlate TILs with clinical, histopathologic, and genomic data in order to (5) help clinical researchers interpret evaluate TILs as a biomarker in cancer and (6) motivate pathologists to use Tumor-TIL maps in daily practice to routinely evaluate TILs in every type of cancer.

Since our study encompasses the use of computational histopathology, immunohistochemistry (IHC), and genomics, we focused on investigating how molecular subtype and estrogen receptor status influence the prognostic value of TILs and spatial features to predict survival and risk of recurrence in the basal (TNBC), HER2, and LumB subgroups. Thus, being able to associate a significantly elevated likelihood of recurrence with TIL infiltrate percentage and two or more “high-risk” spatial features in Tumor-TIL maps of breast cancer from TCGA BRCA and UNC CBCS represent important clinically useful findings. We hope that pathologists will be motivated to use Tumor-TIL maps to evaluate the global abundance and spatial distribution of TILs en route to evaluating sTILs, which could serve as a potential solution to help address high observer variability and low inter-pathologist concordance [82,83].

Even though pathologists generally note the presence and relative magnitude of inflammatory responses after describing the histopathologic features of cancer in tissue specimens, it is a challenging endeavor to just focus on evaluating the nuances of the abundance and spatial distribution of TILs at the tissue level. Not only are pathologists limited by the field of view of microscope objectives, but they would also need to evaluate numerous fields at multiple magnifications to create mental representations resembling Tumor-TIL maps for each case. One could potentially annotate glass slides or WSIs as well, but it would extremely time consuming, cost prohibitive, and limited in terms of the number of cases that could be manually examined. Furthermore, the issues of intra- and interobserver variability would remain unresolved for the most astute pathologist in terms of how TIL infiltrates are examined, classified, and scored. In comparison, our algorithmic approaches uniformly analyze thousands of WSIs and generate Tumor-TIL maps to provide immediate insight about the magnitude and spatial distribution of TILs within the tumor microenvironment.

Furthermore, the spatial features of TIL infiltrates provide insight into the heterogeneity of tumor immunogenicity. Related research in lung adenocarcinoma utilized machine learning to map spatial histology with tumor segmentation and cell segmentation in WSIs, which was integrated with multiregion exome and RNA-sequencing (RNA-seq) data to study geospatial immune variability [84]. High geospatial immune variability was observed between tumor regions within each patient that was not associated with pathologic stage in a sizable study of 970 patients, which supports our observations in Tumor-TIL maps of breast cancer in TCGA BRCA and UNC CBCS. Interestingly, more than one immune cold region in tumors was associated with a higher risk of relapse, independent of tumor size, stage and number of samples analyzed per patient. In addition, low clonal neoantigen burden was observed in tumor regions with decreased lymphocyte accumulation in tumor adjacent stroma, which was hypothesized to be associated with immune-evading subclones and aggressive clinical phenotypes [84].

Limitations of our study include not being able to investigate the association between computed TIL infiltrate percentage and spatial features with histopathologic parameters such as tumor grade due to incomplete data availability. We also could not correlate and compare the association of TILs with chemotherapeutic treatment response in the TCGA BRCA and UNC CBCS cohorts. We also describe both continuous and categorical mechanisms of defining TIL-associated risk. While the continuous computed TIL infiltration score appears to provide finer resolution, it may be more susceptible to noise from tumor burden due to how it is calculated. The spatial features describe several characteristics of TIL infiltrates per entire WSI, which are then grouped into a single categorical variable of multiple “high-risk” features to increase the sensitivity of the metric.

Other limitations of this study include computing TIL infiltrate percentage and scoring high-risk features from only one representative diagnostic WSI per patient. In surgical pathology laboratories, multiple tissue sections are evaluated to classify, grade, and stage each case of cancer, where the most representative slides are typically used for IHC and molecular testing. In future work, we intend to analyze multiple diagnostic WSIs per case in an effort to comprehensively assess the prognostic capability of the abundance and spatial features of TILs to predict high-risk features that were not captured in this study.

The strength of our study is firmly rooted in being able to use the TCGA BRCA and UNC CBCS datasets investigate how the global properties of TILs is associated with clinical outcomes despite several differences in patient demographics and disease states, which we were able to interpret within the context of other studies of TILs in breast cancer. For example, increased TIL infiltration was associated with treatment response in the neoadjuvant setting in all molecular subtypes and longer survival in Her2 and TNBC in a pooled study of several thousands of patients [47]. Notably, increased TILs served as an adverse prognostic factor for survival in luminal Her2 negative breast cancer, indicating the need to better understand the immunogenicity of breast cancer [47]. In a separate study in early breast cancer, high TILs were associated with negative prognostic parameters like increased mitotic activity/high Ki-67 proliferation and negative hormone receptor status while being associated with favorable 5-year disease-free survival (DFS) in hormone receptor positive/Her2-negative patients [85].

Therefore, we intend to use Tumor-TIL maps to guide the development of more sophisticated methods for advanced data mining efforts to explore the relationship of TILs with ductal histology, nuclear size (grade), and mitotic activity/Ki-67 proliferation in different histologic and molecular types of breast cancer [20,42,86,87,88,89,90]. We will also explore whether we can utilize the global abundance and spatial features of TILs to predict the composition of TILs in terms of functional subtypes of T cells, B cells, monocytes, and NK/NKT cells through correlation with IHC, transcriptomic, and methylomic data [5,20,47,78,91,92,93,94,95]. For example, we can envision using spatial features such as the presence of tertiary lymphoid aggregates to investigate the role of humoral responses in breast cancer and predict subtle alterations of TILs in terms of proportions of B cells and T cells [78,93,95,96].

After characterizing these basic relationships of TILs and their spatial features in the TCGA BRCA and UNC CBCS cohort, additional future directions will include exploring how the exquisite diversity of tumor immune interactions in breast cancer can be utilized to study tumor heterogeneity, heterogeneous immunogenicity, and the dynamics of phenotypic plasticity [8]. We also need to explore how Tumor-TIL maps can be used to elucidate the highly complex relationship between the magnitude and spatial distribution of TIL infiltrates with tumor mutational burden (TMB), chromosomal instability (CIN), expression of neoantigens, and treatment response [9,97,98,99,100,101,102,103,104]. We also plan to develop automated methods to computationally define spatial features by using traditional image analysis. After determining the clinical significance of spatial features of TIL infiltrates in this study, further automation of this pipeline will include computationally classifying spatial features in future TIL analyses alongside enabling the integration of other modalities to characterize TILs to support precision oncology applications in breast cancer and other solid tumors.

## 5. Conclusions

We use computational pathology to recapitulate and refine observations about the association of intratumoral lymphocytic infiltrates with prolonged survival in cancer patients, which were first reported a century ago. We present our findings to help achieve greater market penetration for image analysis, machine learning, and computer vision methodology in translational biomedical research and diagnostic pathology. This study demonstrates the use of simple computational pathology tools and statistical analyses in characterizing the utility of TILs as a biomarker to predict clinical outcomes such as survival and risk of recurrence in two special cohorts of breast cancer patients. We also show the value of borrowing concepts and terminology from ecology to describe TILs in a manner that complements the practice of pathology and support the evaluation of TILs as a biomarker. In terms of Pathomics methodology, tumor detection and segmentation are essential tasks for pathologists who microscopically examine histologic tissue samples for cancer classification, grading, and staging. Similarly, classifying lymphocytes is also part of the pathologist’s toolkit when evaluating inflammatory responses. Thus, we hope that showing the ability to predict survival and risk of recurrence by using pathologist inspired Pathomics tools to evaluate TIL infiltrates in two valuable cohorts of breast cancer patients will motivate further research in tumor immunology. While we are not the first group to demonstrate the impact of lymphocytic infiltrates on prognosis, we hope that others might consider implementing similar Pathomics methodology to formally investigate TILs in breast cancer and other solid tumors within the context of the guidelines of the TIL Working Group.

## Figures and Tables

**Table 1:** Comparison of clinical features for TCGA BRCA and UNC CBCS.

Variation	TCGA BRCA (*n* = 935)	UNC CBCS (*n* = 1081)	*p*-Value
Race			
African American	140 (15.0%)	577 (53.4%)	<0.001
Non-African American	711 (76.0%)	504 (46.6%)	
Missing	84 (9.0%)	0 (0%)	
Stage			
Stage I	162 (17.3%)	417 (38.6%)	<0.001
Stage II	545 (58.3%)	499 (46.2%)	
Stage III	213 (22.8%)	138 (12.8%)	
Stage IV	15 (1.6%)	27 (2.5%)	
Grade			
1	N/A	199 (18.4%)	N/A
2	N/A	377 (34.9%)	
3	N/A	478 (44.2%)	
Missing	935 (100%)	27 (2.5%)	
ER by IHC			
Negative	197 (21.1%)	249 (23.0%)	0.676
Positive	692 (74.0%)	831 (76.9%)	
Missing	46 (4.9%)	1 (0.1%)	
PAM50 Subtype			
Basal	167 (17.9%)	245 (22.7%)	0.0383
Her2	76 (8.1%)	82 (7.6%)	
LumA	489 (52.3%)	553 (51.2%)	
LumB	203 (21.7%)	201 (18.6%)	
Percent Infiltration (%)			
Mean (SD)	7.7 (10.4)	5.6 (10.2)	<0.001
Median [Min, Max]	3.8 [0, 64.2]	1.9 [0, 88.9]	
TIL Class			
Low Infiltration	648 (69.3%)	806 (74.6%)	0.01
High Infiltration	287 (30.7%)	275 (25.4%)	
“TIL Sensitive”			
No	477 (51.0%)	599 (55.4%)	0.217
Yes	430 (46.0%)	481 (44.5%)	
Missing	28 (3.0%)	1 (0.1%)	
Progression Events			
Number with Event	119 (12.7%)	164 (15.1%)	0.131
Median days to event [Min,Max]	791 (21,5117)	1048.5 (161,3274)	0.0288

**Table 2:** Descriptions of spatial features of Tumor-TIL maps and scoring criteria. Representative images for each score and category are shown in Figure 3.

Scoring Category	Score Range	Score	Criteria
Intratumoral Strength	0–3	0	No patches positive for TILs present within the tumor region
		1	Low intratumoral TIL infiltration (weak) with scant/limited TIL-positive patches within tumor region
		2	Intermediate level of TIL infiltration (moderate) with variable spatial distribution (e.g., high density of TILs in some regions and no TILs in others, but not limited to a single focus)
		3	High intratumoral TIL infiltration (strong) with numerous and diffuse TIL-positive patches
Intratumoral Deserts	0–1	0	small regions of tumor without TIL patches (absent) OR focal areas tumor with little to no TIL patches (weak) (≤10% of tumor area)
		1	significant intratumoral regions completely clear of TIL infiltrate (≥25% of tumor area)
Intratumoral Forests	0–1	0	TIL infiltrate either not present (absent) or TIL-positive patches are evenly distributed alongside TIL-negative tumor patches (weak) with 1–2 small confluent groups of TILs
		1	Confluent groups of TILs spanning ≥10% of tumor area present (strong)
Peritumoral Strength	0–3	0	No patches positive for TILs present in the region of the invasive boundary (absent)
		1	Low peritumoral TIL infiltration with scant/scattered TIL-positive patches at the boundary of tumor and adjacent normal tissues (weak)
		2	Intermediate level of TIL infiltration (moderate)
		3	High peritumoral TIL infiltration (marked) with nearly confluent accumulation of TIL-positive patches surrounding the tumor at the invasive boundary
Tertiary Lymphoid Aggregates	0–1	0	No significant presence of TIL clusters or minimal focal aggregates beyond the peritumoral region
		1	Multifocal lymphoid aggregates exist beyond the peritumoral region

**Table 3:** Comparison of spatial features in the “TIL sensitive” cohorts of TCGA BRCA and UNC-UNC CBCS.

Feature	TCGA BRCA (*n* = 430)	UNC CBCS (*n* = 481)	*p*-Value
Percent Infiltration (%)			
Mean (SD)	10 (11.9)	7.89 (12)	0.00725
Median [Min, Max]	5.4 [0, 64.2]	3.3 [0, 88.9]	
TIL Class			
Low	247 (57.4%)	298 (62.0%)	0.187
High	183 (42.6%)	183 (38.0%)	
Intratumoral Strength			
0	4 (0.9%)	11 (2.3%)	<0.001
1	247 (57.4%)	282 (58.6%)	
2	113 (26.3%)	64 (13.3%)	
3	42 (9.8%)	34 (7.1%)	
Unscored	24 (5.6%)	90 (18.7%)	
TIL Forests			
Absent	188 (43.7%)	260 (54.1%)	<0.001
Present	218 (50.7%)	131 (27.2%)	
Unscored	24 (5.6%)	90 (18.7%)	
TIL Deserts			
Absent	260 (60.5%)	191 (39.7%)	<0.001
Present	146 (34.0%)	200 (41.6%)	
Unscored	24 (5.6%)	90 (18.7%)	
Peritumoral Strength			
0	9 (2.1%)	3 (0.6%)	0.365
1	149 (34.7%)	138 (28.7%)	
2	146 (34.0%)	149 (31.0%)	
3	102 (23.7%)	101 (21.0%)	
Unscored	24 (5.6%)	90 (18.7%)	
Peritumoral Aggregates			
Absent	87 (20.2%)	10 (2.1%)	<0.001
Present	319 (74.2%)	381 (79.2%)	
Unscored	24 (5.6%)	90 (18.7%)	

**Table 4:** Univariate hazard ratios for spatial features.

Metric	Level	TCGA BRCA Hazard Ratio	UNC CBCS Hazard Ratio	High-Risk Feature (Score)
Intratumoral strength	1	0.62	0.69	Low intratumoral strength (0–1)
	2	0.27	0.34	
	3	0.19	0.28	
TIL deserts	Present	2.0 **	1.2	TIL deserts present (1)
TIL forests	Present	0.56 *	0.65	TIL forests absent (0)
Peritumoral strength	1	2.07	0.49	Low peritumoral strength (0–1)
	2	1.14	0.41	
	3	0.61	0.17 *	
Lymphoid aggregates	Present	0.78	0.42	Lymphoid aggregates absent (0)

** = *p* < 0.05, * = *p* < 0.01.

## Data Availability

Lymphocyte detection and breast cancer segmentation models, data, and TIL maps are available in previously published Github repositories [14,15,17].

## Supplementary Materials

The following supporting information can be downloaded at: https://www.mdpi.com/article/10.3390/cancers14092148/s1, Figure S1: Representative Tumor-TIL maps demonstrating variations in TIL deserts, forests, and lymphoid aggregates. Figure S2: Spatial features provide information about spatial distribution of TILs beyond percent infiltration. Figure S3: Multivariate analyses of spatial features and overall TIL invasion provide similar information.
