# Supplementary material for: Spatial Characterization of Tumor-Infiltrating Lymphocytes and Breast Cancer Progression
Source: Cancers (Basel). 2022 Apr 26;14(9):2148. doi: 10.3390/cancers14092148 (PMC9105398; doi:10.3390/cancers14092148)
Supplement: Supplementary file 1 [file cancers-14-02148-s001.zip › cancers-1662627-supplementary.pdf]

cancers MDPI

Supplemental:

Table S1. Tumor TIL composite images demonstrating representative images of spatial features

Score	0	1	2	3
Intratumoral strength	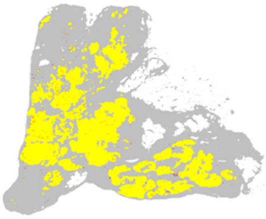	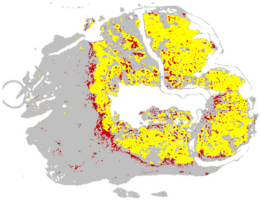	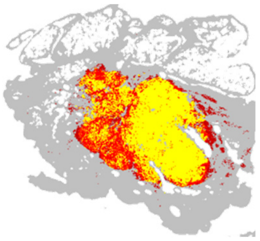	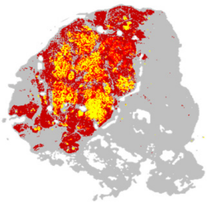
Peritumoral strength	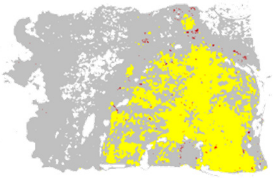	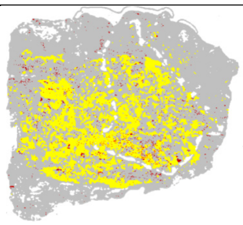	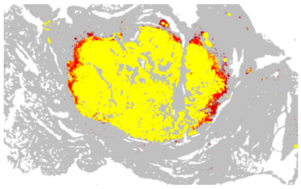	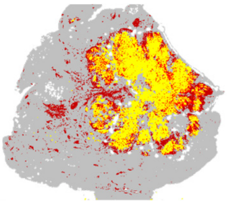
TIL deserts	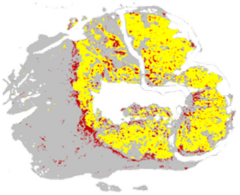	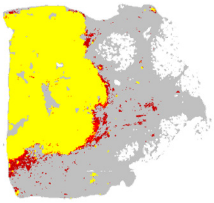		
TIL forests	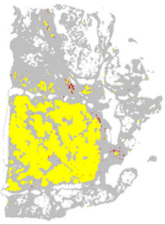	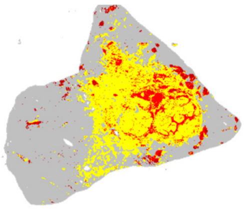		
Lymphoid aggregates	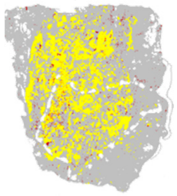	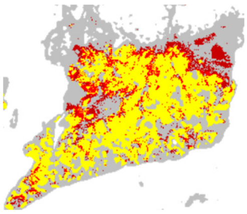		

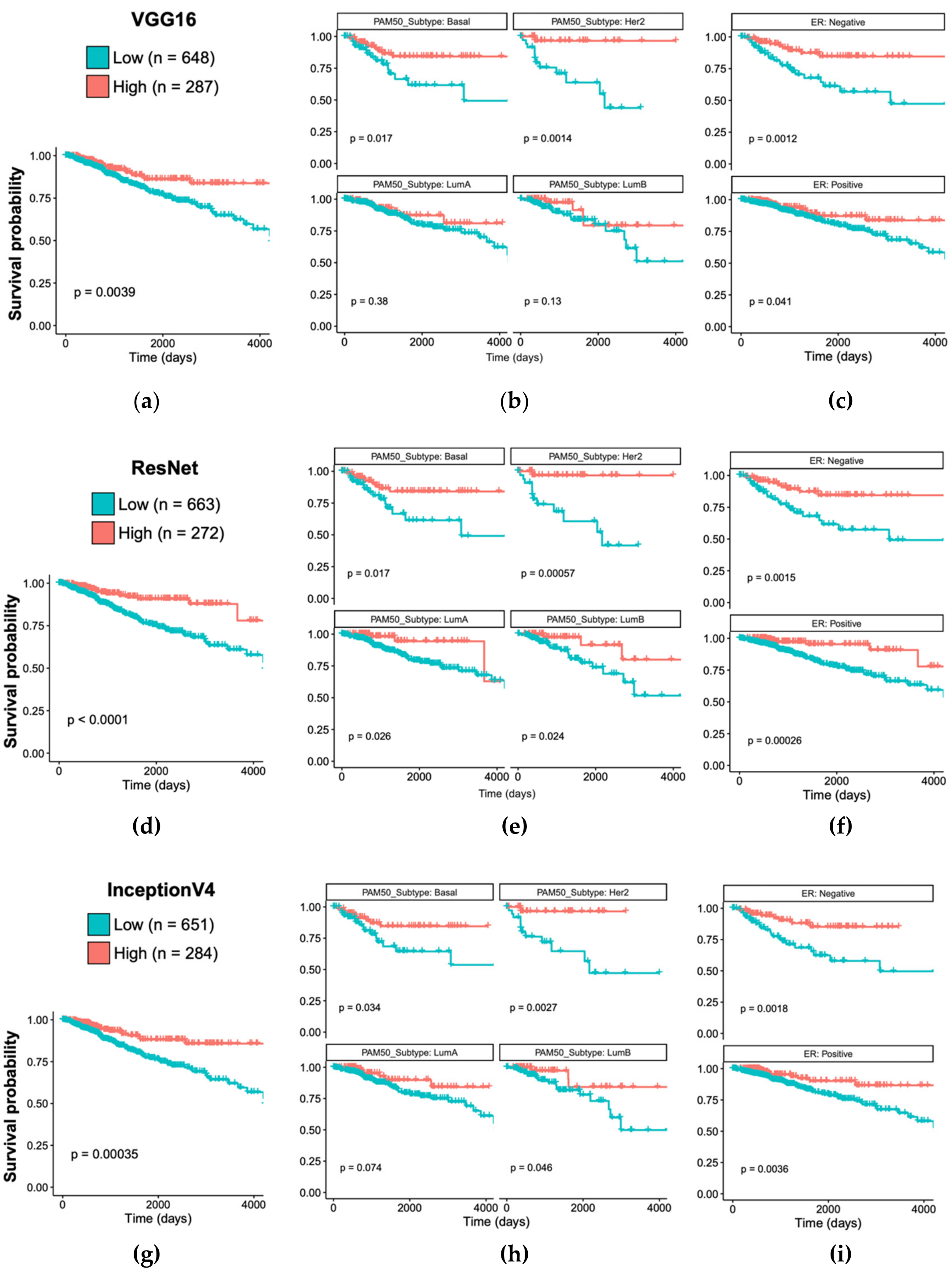

Figure S1. *Kaplan-Meier (KM) plots of proportion of patients yet to progress based on tumor infiltrating lymphocyte (TIL) class.* Distinct TIL models: VGG (a-c), ResNet (d-f), and InceptionV4(g-i) were used to detect TILs, percent infiltration was calculated, and TIL infiltration class was determined as high or low based on being above or below the mean percent infiltration for the given algorithm. KM plots are shown for the entire TCGA cohort (a,d,g), split by PAM50 molecular subtype (b,e,h), and split by estrogen receptor status (c,f,g).

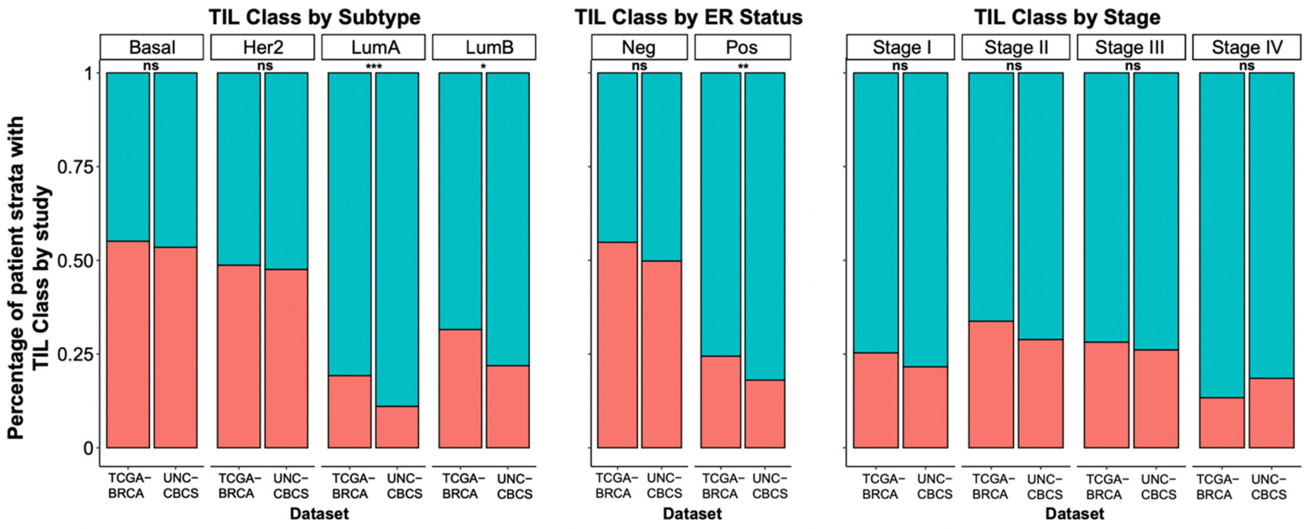

(**a**) (**b**) (**c**)

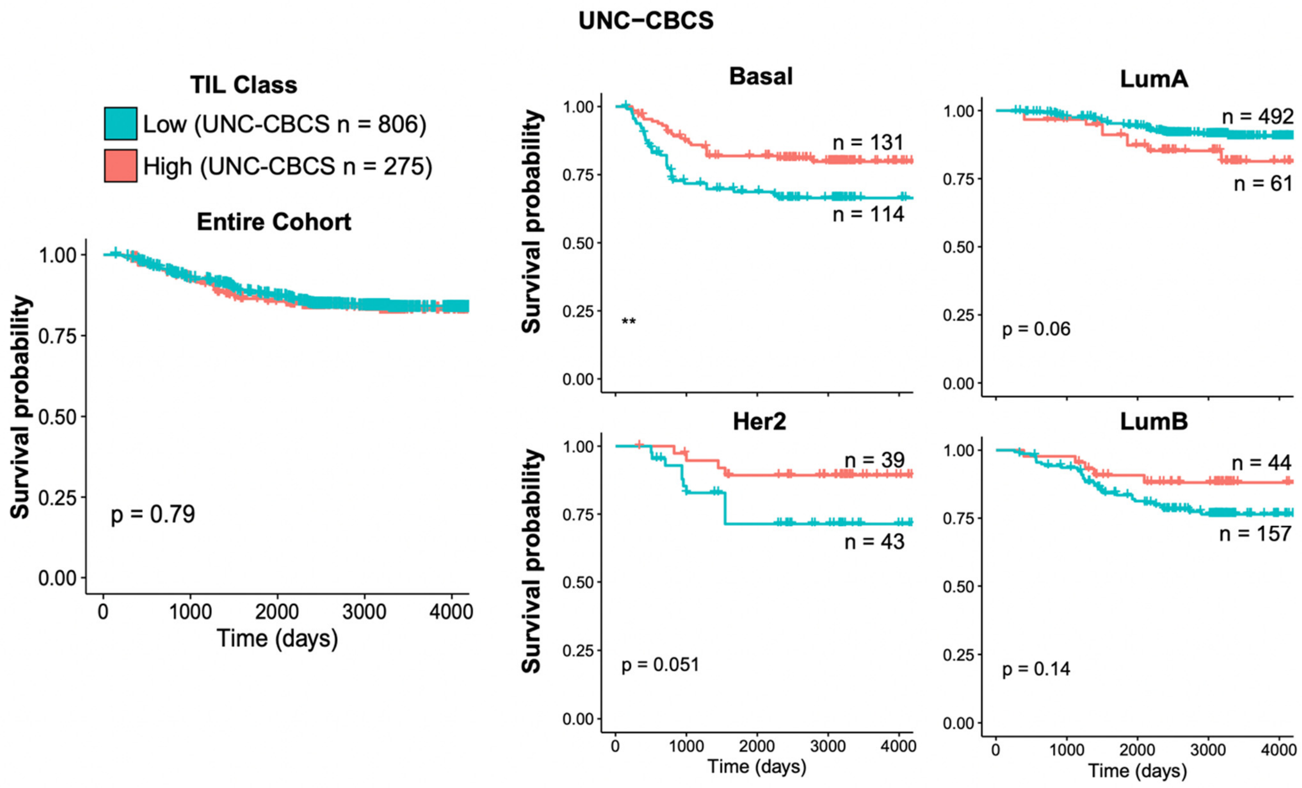

(**d**) (**e**)

Figure S2. *TIL composition and relationship to progression free interval (PFI).* Median percent infiltration of each study was used to distinguish cases as falling into high or low TIL class; percent infiltration was determined using VGG TIL detection algorithm. (a-c) Percentage of patient strata classified as high and low TILs is shown for the TCGA-BRCA and UNC-CBCS studies grouped by: PAM50 molecular subtype (a), estrogen receptor (ER) status (b), and tumor stage (c). (d-e) Kaplan-Meier plots of proportion of cohort yet to progress after dividing patients into high and low TIL infiltration groups around the mean percent infiltration for the entire UNC-CBCS cohort (d), and with cases split by PAM50 molecular subtype (e).
